# Impact of Temperature and Nutrients on Carbon: Nutrient Tissue Stoichiometry of Submerged Aquatic Plants: An Experiment and Meta-Analysis

**DOI:** 10.3389/fpls.2017.00655

**Published:** 2017-05-04

**Authors:** Mandy Velthuis, Emma van Deelen, Ellen van Donk, Peiyu Zhang, Elisabeth S. Bakker

**Affiliations:** ^1^Department of Aquatic Ecology, Netherlands Institute of EcologyWageningen, Netherlands; ^2^Department of Biology, Utrecht UniversityUtrecht, Netherlands

**Keywords:** submerged freshwater and marine macrophytes, meta-analysis, microcosm experiment, *Elodea nuttallii*, eutrophication, global warming, carbon:nutrient stoichiometry, growth rate

## Abstract

Human activity is currently changing our environment rapidly, with predicted temperature increases of 1–5°C over the coming century and increased nitrogen and phosphorus inputs in aquatic ecosystems. In the shallow parts of these ecosystems, submerged aquatic plants enhance water clarity by resource competition with phytoplankton, provide habitat, and serve as a food source for other organisms. The carbon:nutrient stoichiometry of submerged aquatic plants can be affected by changes in both temperature and nutrient availability. We hypothesized that elevated temperature leads to higher carbon:nutrient ratios through enhanced nutrient-use efficiency, while nutrient addition leads to lower carbon:nutrient ratios by the luxurious uptake of nutrients. We addressed these hypotheses with an experimental and a meta-analytical approach. We performed a full-factorial microcosm experiment with the freshwater plant *Elodea nuttallii* grown at 10, 15, 20, and 25°C on sediment consisting of pond soil/sand mixtures with 100, 50, 25, and 12.5% pond soil. To address the effect of climatic warming and nutrient addition on the carbon:nutrient stoichiometry of submerged freshwater and marine plants we performed a meta-analysis on experimental studies that elevated temperature and/or added nutrients (nitrogen and phosphorus). In the microcosm experiment, C:N ratios of *Elodea nuttallii* decreased with increasing temperature, and this effect was most pronounced at intermediate nutrient availability. Furthermore, higher nutrient availability led to decreased aboveground C:P ratios. In the meta-analysis, nutrient addition led to a 25, 22, and 16% reduction in aboveground C:N and C:P ratios and belowground C:N ratios, accompanied with increased N content. No consistent effect of elevated temperature on plant stoichiometry could be observed, as very few studies were found on this topic and contrasting results were reported. We conclude that while nutrient addition consistently leads to decreased carbon:nutrient ratios, elevated temperature does not change submerged aquatic plant carbon:nutrient stoichiometry in a consistent manner. This effect is rather dependent on nutrient availability and may be species-specific. As changes in the carbon:nutrient stoichiometry of submerged aquatic plants can impact the transfer of energy to higher trophic levels, these results suggest that eutrophication may enhance plant consumption and decomposition, which could in turn have consequences for carbon sequestration.

## Introduction

Human activity has led to rapid environmental changes on our planet (Vitousek et al., 1997; Steffen et al., 2015). Water temperatures in marine and freshwater systems have increased over the last decades and are expected to increase further over the course of the century (Mooij et al., 2008; Adrian et al., 2009; IPCC, 2014). Furthermore, agriculture and industrialization have a strong impact on nutrient cycles (Carpenter et al., 1998; Tilman et al., 2001) and are major sources of nitrogen and phosphorus input in freshwater and marine ecosystems. Changes in temperature and nutrient availability can have consequences for the abundance of submerged aquatic plants that occur in the shallow parts of aquatic ecosystems (Bornette and Puijalon, 2011).

Changes in plant abundances and growth rates can have effects on their nutrient demand and uptake and as such can influence their carbon:nutrient stoichiometry (Sterner and Elser, 2002). Alterations in internal stoichiometry in turn can have consequences for ecosystem functioning, as lower carbon:nutrient ratios can make aquatic plants more palatable to herbivores (Dorenbosch and Bakker, 2011), resulting in higher herbivory rates and stimulated top-down control (Olsen and Valiela, 2010; Bakker and Nolet, 2014) and leading to lowered carbon stocks in the form of plant biomass (Heithaus et al., 2014; van Altena et al., 2016).

However, contrasting hypotheses exist on how temperature and nutrient availability may affect carbon:nutrient ratios in aquatic plants. Elevated temperature can lead to an increase in plant biomass and a biomass dilution effect, where increased growth rates are accompanied by reduced tissue content (per unit of biomass) of a particular element (Taylor et al., 1991; Vermaat and Hootsmans, 1994). In terrestrial plant and phytoplankton research, this effect is referred to as enhanced nutrient-use efficiency (An et al., 2005; De Senerpont Domis et al., 2014). According to this hypothesis, elevated temperature would lead to reduced N and P content in aquatic plants and a subsequent increase in carbon:nutrient ratios. Alternatively, higher temperatures can increase the rate of cellular processes, but do not necessarily lead to an unbalanced nutrient uptake, provided that enough nutrients are available in the environment, and therefore would not result in changes in carbon:nutrient ratios.

Similarly, nutrient addition can positively affect the nutritional quality of aquatic plants (e.g., lower carbon:nutrient ratios; Burkholder et al., 2007; Bakker and Nolet, 2014) as they may take up relatively more nutrients compared to carbon. This fertilization effect is demonstrated for terrestrial plants in a recent meta-analysis (Sardans et al., 2012). Furthermore, the combined effect of elevated temperature and nutrient addition may be antagonistic under the hypotheses of enhanced nutrient-use efficiency and luxurious uptake, as the former would be expected to increase carbon:nutrient ratios, while the latter would decrease carbon:nutrient ratios.

Here, we aimed to quantify the effects of temperature and nutrient addition on the carbon:nutrient stoichiometry of submerged aquatic angiosperms. We hypothesized that (1) both elevated temperature and nutrient addition lead to enhanced growth rates of submerged angiosperms, (2) if the biomass-dilution effect applies, elevated temperature will lead to higher carbon:nutrient ratios, whereas (3) nutrient addition is expected to lead to decreased carbon:nutrient ratios. These hypothesized changes in carbon:nutrient ratios are expected to be driven by changes in nutrient contents as opposed to carbon (4). Furthermore, we hypothesized that elevated temperature and nutrient addition are antagonists in their combined effect on carbon:nutrient ratios (5).

We tested these hypotheses using two complementary approaches. First, we performed a full-factorial experiment on the effects of temperature and sediment nutrient content (and their interaction) on the growth and carbon:nutrient stoichiometry of the submerged freshwater angiosperm *Elodea nuttallii*. *E. nuttallii* is native to North America, but has become common throughout the northern hemisphere in the 1900s (Cook and Urmi-König, 1985). Subsequently, a meta-analytic approach was used to address the effect of elevated temperature and nutrient addition on submerged angiosperms in general. We performed a meta-analysis using experimental studies that simulated temperature rise and/or increased nutrient (nitrogen and phosphorus) input and documented the effects on plant growth and carbon:nutrient stoichiometry. In this analysis, we included both marine and freshwater plants. Whereas the responses of aquatic plants to environmental change in marine and freshwater systems are mostly discussed independently, we expected that responses in growth and carbon:nutrient stoichiometry similarly apply to both submerged marine and freshwater angiosperms alike.

## Materials and methods

### *Elodea* laboratory experiment

#### Experimental set-up

To test the effect of temperature and sediment nutrient content on *Elodea nuttallii*, a full-factorial microcosm experiment was set up. Shoots of *E. nuttallii* were collected from a small pond on the grounds of The Netherlands Institute of Ecology (NIOO-KNAW), Wageningen, The Netherlands (51°59′15.0″N; 5°40′14.8″E) on 07-09-2015. After collection, the plants were rinsed and acclimatized at room temperature for 2 days prior to the start of the experiment.

The experiment was carried out in 4 L plastic microcosms (14 × 14 × 21 cm), which contained 1.1 L of sediment and 2.7 L of water. Nutrient treatments were achieved by mixing artificial pond sediment (20% organic matter, Velda, Enschede, The Netherlands) with sand and consisted of 12.5, 25, 50, and 100% (v/v) of pond sediment (*n* = 5), covered with a one centimeter layer of sand. The artificial pond sediment contained 31 ± 1.8, 0.80 ± 0.048, and 0.11 ± 0.0084% (mean ± SE) C, N and P respectively. One shoot fragment of *E. nuttallii* of ± 5.5 cm (C:N = 19 ± 1.4, C:P = 435 ± 72; mol:mol; *n* = 5) was placed in the middle of each microcosm and the microcosm was topped off with nutrient-poor tap water [3.5 ± 0.5 (mean ± SE) μM DIN and undetectable levels of DIP]. The microcosms were placed in four aquaria, which served as temperature-regulated water baths. The temperature treatments were 10, 15, 20, and 25°C, which were obtained by a computer-controlled (Specview 32/859, SpecView Ltd., Uckfield, UK) custom-made climate control system. These temperatures are within the range of natural temperatures *E. nuttallii* would encounter, as water temperatures in the Netherlands vary seasonally between 4 and 23°C (van Dam, 2009). Light (14:10 hours light:dark) was provided by two 28W TL5 HE lamps (Philips, Eindhoven, The Netherlands), hung above the aquaria with an average light intensity at the water surface of 30 μmol s^−1^ m^−2^. To ensure equal light conditions between treatments, position of the microcosms in the water bath was randomized once a week and evaporation losses were compensated by additions of demi-water. To prevent excessive periphyton and phytoplankton growth during the experiment, one periphyton-grazing snail (*Planorbarius corneus*) and one filtering mussel (*Dreissena polymorpha*) were put in each microcosm. In pilot tests, *P. corneus* did not feed on *E. nuttallii* (Peiyu Zhang, personal observation) and no grazing on the plants was observed during the experiment. The snails were retrieved from the same pond as the plants, and the mussels were collected from the Nether Rhine, Wageningen, the Netherlands (51°57′12.9″N 5°39′48.2″E). In case either snail or mussel died, another one was added.

#### Harvest

After 58 days the experiment was terminated. From the middle of each microcosm, water samples were taken to determine dissolved nutrient concentrations, filtered over prewashed GF/F filters (Whatman, Maidstone, U.K.) and stored at −20°C until further analysis. Samples for pore water nutrients were taken in each microcosm through a 10 cm Rhizon SMS (Rhizosphere, Wageningen, the Netherlands) and stored at −20°C until further analysis. Plants were cut at the sediment level, and the above- and belowground biomass was harvested and rinsed with demi-water. All plant materials (above- and belowground) were dried at 60°C until constant dry mass and weighed. During the harvest, basic parameters were measured that describe the growing conditions (pH, alkalinity and seston chlorophyll-a). Methods and results of these measurements can be found in Supplementary Material S1.

#### Chemical analysis

Plant material was grinded to a fine powder on a microfine grinder (MF 10 basic, IKA-werke, Staufen, Germany) or in test tube with a 1/8” ball bearing (Weldtite, Lincolnshire, UK) on a Tissuelyser II (QIAGEN, Germantown, USA). For nitrogen (N) and carbon (C) content, 0.2–2 mg dry mass was analyzed on a NC analyser (FLASH 2000 NC elemental analyser, Brechbueler Incorporated, Interscience B.V., Breda, The Netherlands). For phosphorus (P) content, 1–4 mg dry mass was combusted in a Pyrex glass tube at 550°C for 30 min. Subsequently, 5 mL of persulfate (2.5%) was added and samples were autoclaved for 30 min at 121°C. Digested P (as ${\text{PO}}_{4}^{3-}$) was measured on a QuAAtro39 Auto-Analyzer **(**SEAL Analytical Ltd., Southampton, U.K.). Concentrations of dissolved nutrients (${\text{PO}}_{4}^{3-}$, ${\text{NO}}_{2}^{-}$, ${\text{NO}}_{3}^{-}$ and ${\text{NH}}_{4}^{+}$) of thawed water-samples were determined on a QuAAtro39 Auto-Analyzer (SEAL Analytical Ltd., Southampton, U.K.). Results for the dissolved nutrients in the water column can be found in Figure S1.2.

#### Calculations and statistics

Plant specific growth rate (SGR) was calculated with the following formula:

$$\begin{array}{l} SGR\;=\;\frac{Ln\left(DW_{t}\right)\;-\;Ln\left(DW_{0}\right)}{t} \end{array}$$

Where *DW*_*t*_ is the plant aboveground dry weight at the end of the experiment, *DW*_0_ the dry weight at the beginning of the experiment (determined by multiplying the initial wet weight with the plants wet weight/dry weight ratio) and *t* the experimental duration (=58 days).

Data on above- and belowground parameters (specific growth rate, above- and belowground biomass, carbon:nutrient stoichiometry and elemental contents) and dissolved nutrient concentrations (DIN and DIP) in the water column and the pore water were tested for effects of temperature, nutrients and their interaction with generalized linear models (function *glm* from stats package). Visual examination of the data distribution (function *hist*) led to the use of a gamma distribution. *Post-hoc* tests within treatment levels were carried out using Tukey contrasts [function glht from multcomp package (Hothorn et al., 2008)], with *P*-values corrected for multiple comparison as described by Benjamini and Hochberg (1995).

### Meta-analysis

#### Systematic literature review and data collection

A systematic literature review was carried out in Web of Science based on the guidelines described by the Collaboration for Environmental Evidence (2013). The search term (“submerged macrophyte^*^” OR “aquatic plant” OR isoetid OR macrophyte^*^ OR “aquatic weed” OR seagrass^*^) AND (stoichiometr^*^ OR “^*^chemical composition” OR “nutritional quality” OR “nutrient composition” OR “elemental composition” OR “nutrient content” OR “nutrient ratio^*^” OR C:N OR C:P OR N:P OR “plant nutrient concentration^*^”) AND (warming OR eutrophication OR temperature^*^ or enrichment or fertilis^*^ or “nutrient availability”) on 01-11-2016 gave 414 hits. Further selection based on abstracts, graphs and tables led to 47 papers that contained information on temperature and/or nutrient effects on elemental composition of submerged angiosperms. Data originating from light limited conditions (as indicated in the paper itself) were excluded from analysis, as well as studies without reported standard errors or deviations, and studies with limited (*n* < 2) or non-reported sample size. From the selected papers, data on C:N and C:P ratios were extracted with use of Plotdigitizer and Engauge and converted to molar ratios when necessary. In addition, C, N and P contents, growth rates (above- or belowground), habitat (marine or freshwater), which part of the plant was analyzed (above- or belowground) and sample size were extracted when reported. If the described methodology indicated possible additional results that were not reported, corresponding authors were contacted to retrieve those data. If experiments reported several measurements over time, only the final measurement was extracted. If papers contained multiple experiments, on the same or on different species, these were extracted as being separate studies.

#### Data selection

Control and elevated treatments were defined for both temperature and nutrient addition for each experiment separately. The lowest water temperature reported was defined as the control temperature treatment and 3–6°C above that temperature [equivalent to RCP scenario 8.5 from IPCC (2014)] was defined as the elevated temperature treatment. For the nutrient addition studies, those studies that manipulated both nitrogen and phosphorus simultaneously were selected. The lowest nutrient condition reported was defined as the control treatment and the highest as the elevated treatment. The data was then split up into above- and belowground plant responses, as different parts of plants were expected to respond differently (Bloom et al., 1985). These selection criteria led to a total of 50 studies on nutrient addition spread over 26 papers (of which 50 and 11 on above- and belowground responses respectively) and 3 studies on temperature (only on aboveground responses) originating from 3 papers. Temperature studies were all conducted in mesocosms, whereas nutrient studies included *in situ* fertilization experiments (38), mesocosm experiments (9) and laboratory experiments (3). Of the nutrient addition studies, 9 studies tested a range of nutrient concentrations of which the highest and lowest were selected, while the majority (41) specifically looked at the addition of nitrogen and phosphorus to the system relative to a control level. An overview of the dataset selection can be found in Figure S2 and an overview of the selected papers in Supplementary Material S3.

#### Response factors and statistics

Delta response ratios and their variances were calculated for each separate study according to Lajeunesse (2015):

$$\begin{array}{l} RR\Delta =Ln\left(\frac{Xtreatment}{Xcontrol}\right)\\ \;+\frac{1}{2}\left[\frac{{\left(SDtreatment\right)}^{2}}{Ntreatment*Xtreatment^{2}}-\frac{{\left(SDcontrol\right)}^{2}}{Ncontrol*Xcontrol^{2}}\right]\\ var\left(RR\Delta \right)=\frac{{\left(SDtreatment\right)}^{2}}{Ntreatment*Xtreatment^{2}}+\frac{{\left(SDcontrol\right)}^{2}}{Ntreatment*Xcontrolt^{2}}\\ \;+\frac{1}{2}\left[\frac{{\left(SDtreatment\right)}^{4}}{Ntreatment^{2}*Xtreatment^{4}}+\frac{SDcontrol^{4}}{Ncontrol*Xcontrolt^{4}}\right] \end{array}$$

Where X denotes mean of the fixed factor of interest [C:N and C:P ratio, growth rate (μ) and C, N and P contents], SD the standard deviation of that mean and N the sample size.

All statistics were carried out in R (R Core Team, 2015). To test whether response ratios deviated from zero, mixed effect models were fitted to the response ratios and their variances with the function *rma.mv* [package metafor; Viechtbauer (2010)], incorporating reference and species as random effects. To test whether freshwater and marine systems differed in response ratio, separate models were compared for significant differences between the two habitat types (by adding habitat as a moderator to the function *rma.mv*).

## Results

### *Elodea* experiment

#### Biomass responses

**Figure 1:**
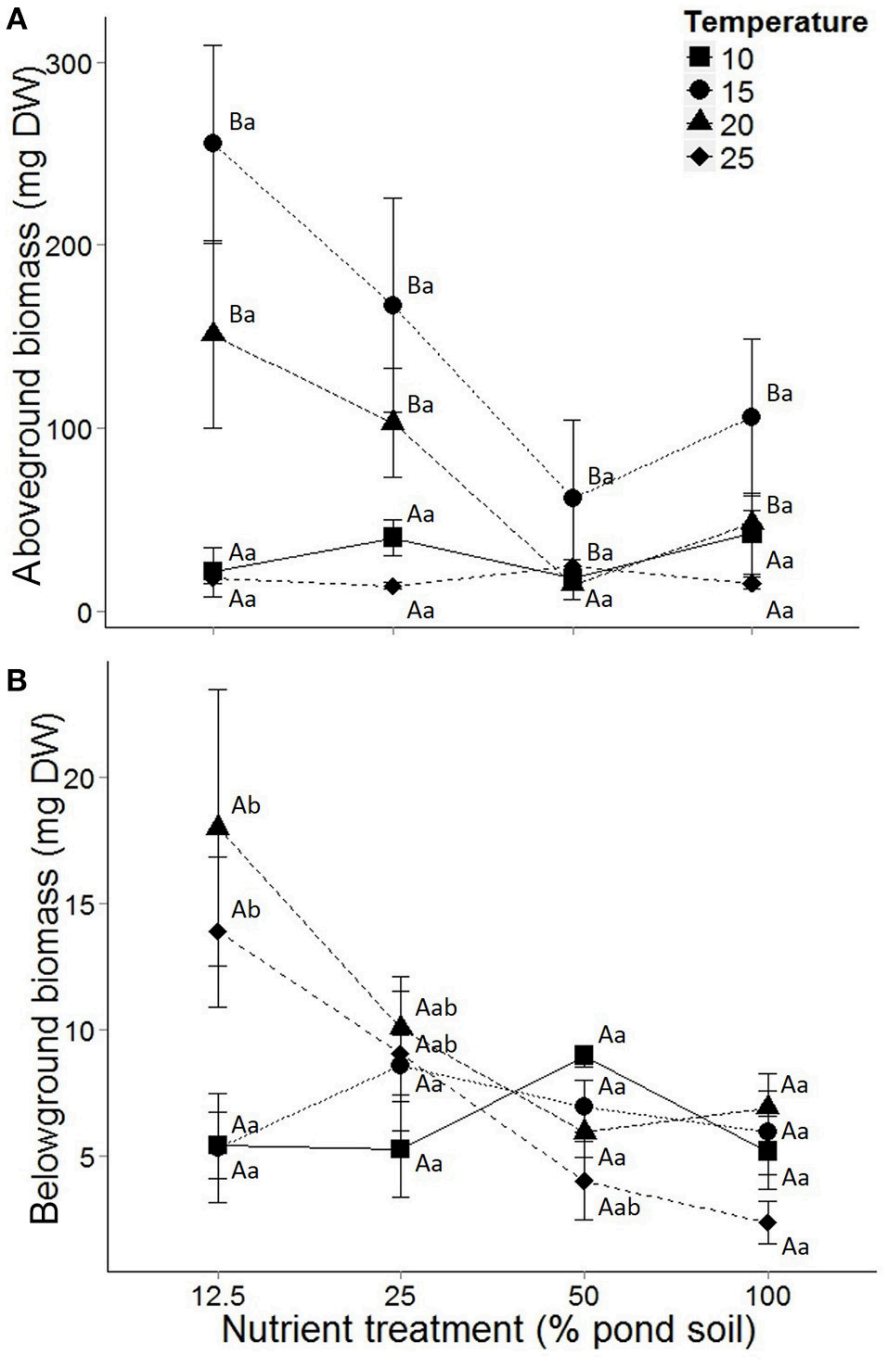
**Above—(A) and belowground **(B)** biomass of *Elodea nuttallii* grown at different temperatures and sediment nutrient content**. Temperature treatments include 10 (■), 15 (•), 20 (▴) and 25 (♦)°C. Dots represent means and error bars standard error of the mean (*n* = 5). Capital and lower case letters indicate *post-hoc* differences between temperature and nutrient treatments, respectively.

Temperature affected the specific growth rate and above- and belowground biomass of *E. nuttallii* (Table 1). As indicated by the interaction term, temperature only affected specific growth rate at intermediate sediment nutrient content (e.g., 25%), with optimal growth at 15 and 20°C (*P* < 0.05, Tukey *post-hoc* comparison; Figure S4). Similarly, aboveground biomass was highest at these temperatures, irrespective of nutrient treatment (*P* < 0.05; Figure 1A). Belowground biomass of *E. nuttallii* was affected by temperature, and this effect interacted with nutrient treatment (Figure 1B, Table 1). The effects of temperature on belowground biomass seemed strongest in the lowest nutrient treatments (e.g., 12.5%), where biomass tended to increase 3-fold between 15 and 20°C but these effects were not significant in *post-hoc* tests (*P* = 0.09).

**Table 1:** **Summary of generalized linear model analysis of the ***Elodea*** experiment, describing the effect of temperature treatment, nutrient treatment and their interaction on the biomass, carbon:nutrient stoichiometry and elemental contents of ***Elodea nuttallii*** and nutrient concentrations**.

		**Chi-square values**		
	**Unit**	**Temperature**	**Nutrients**	**Temperature × Nutrients**
**BIOMASS VARIABLES**				
Specific growth rate	day^−1^	16.7^***^	8.8^*^	7.3
Aboveground biomass	mg DW	62.4^***^	17.7^***^	13.1
Belowground biomass	mg DW	10.8^*^	18.6^***^	20.8^*^
**CARBON:NUTRIENT STOICHIOMETRY**				
Aboveground C:N	mol:mol	43.6^***^	3.5	22.5^**^
Aboveground C:P	mol:mol	7.7	45.6^***^	8.2
Belowground C:N	mol:mol	4.7	8.8^*^	21.8^**^
Belowground C:P	mol:mol	6.0	12.0^**^	7.5
**ELEMENTAL CONTENTS**				
Aboveground C	mol g DW^−1^	5.1	0.7	1.3
Aboveground N	mol g DW^−1^	15.5^**^	1.4	3.1
Aboveground P	mol g DW^−1^	7.6	83.4^***^	45.7^***^
Belowground C	mol g DW^−1^	9.4^*^	35.2^***^	9.8
Belowground N	mol g DW^−1^	7.4	0.9	14.3
Belowground P	mol g DW^−1^	6.1	1.2	17.3^*^
**NUTRIENT CONCENTRATIONS**				
Pore water DIN	μM	42.1^***^	163.9^***^	19.8^*^
Pore water DIP	μM	47.9^***^	317.6^***^	29.3^***^

Significant results are indicated in bold, with

*** *P < 0.001*,

** P < 0.01 and

* *P < 0.05*.

Sediment nutrient content affected the specific growth rate and the above- and belowground biomass of *Elodea nuttallii* (Table 1). However, no significant differences between nutrient treatments could be observed for specific growth rate (Figure S4), nor for aboveground biomass (Figure 1A) in the *post-hoc* comparisons. Belowground biomass decreased with increasing sediment nutrient content, and this effect interacted with temperature treatment (Table 1; Figure 1B). Belowground biomass tended to decrease 6-fold over the entire range of nutrient treatments at 25°C, but this effect was not significant (*P* = 0.08).

#### Carbon:nutrient stoichiometry

**Figure 2:**
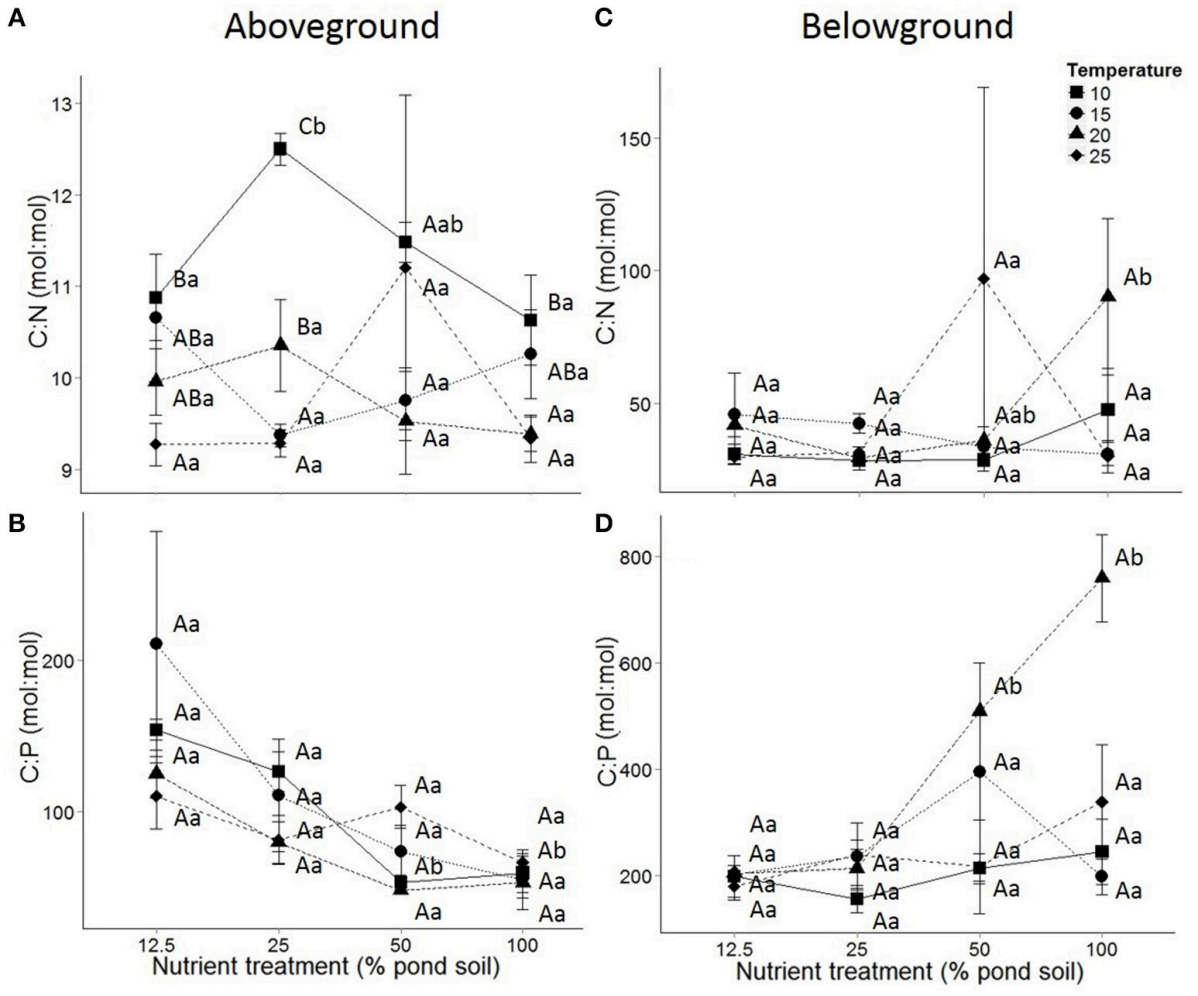
**Above—(A,B) and belowground **(C,D)** carbon:nutrient stoichiometry of *Elodea nuttallii* in response to sediment nutrient content**, with **(A,C)** C:N and **(B,D)** C:P ratios. Temperature treatments include 10 (■), 15 (•), 20 (▴) and 25 (♦)°C. Dots represent means and error bars standard error of the mean. Capital and lower case letters indicate *post-hoc* differences between temperature and nutrient treatments, respectively.

Temperature negatively affected aboveground C:N ratios (Table 1, Figure 2A), which was most visible at intermediate sediment nutrient content (25%). In this treatment, aboveground C:N ratios decreased moderately but significantly between 10 and 25°C (*P* < 0.001, Tukey *post-hoc* comparison). No effects of temperature on aboveground C:P ratios were observed, nor on belowground C:N and C:P ratios (Table 1, Figures 2B–D).

Sediment nutrient content affected aboveground C:P ratio, while no effect on aboveground C:N ratios was observed (Table 1). Aboveground C:P ratios of *E. nuttallii* were negatively affected by increasing sediment nutrient content (Figure 2B, Table 1). This effect was most visible at 15°C, where the C:P ratio significantly decreased 4-fold the entire range of nutrient treatments (*P* < 0.001, Tukey *post-hoc* comparison). Belowground C:N and C:P ratios were affected by nutrient treatment, and the effect on C:N interacted with temperature (Figure 2C). Belowground C:N ratios significantly increased between 25 and 100% nutrient treatments at 20°C, while belowground C:P ratios increased 4-fold between those nutrient treatments at the same temperature (*P* < 0.01; Figure 2D).

#### Elemental contents

Accompanied by the changes in aboveground C:N ratio, temperature seemed to affect aboveground N content (Table 1). However, no differences between any of the temperature treatments could be detected in *post-hoc* comparisons (Figure S5B). Belowground carbon content was affected by temperature, and halved between 10 and 25°C in the lowest sediment nutrient treatments (12.5 and 25%; *P* < 0.05). No effects of temperature on aboveground C and P content were observed, nor on belowground N and P contents (Table 1).

Sediment nutrient content affected aboveground P content, with a 3-fold increase over the entire range of nutrient treatments (*P* < 0.05; Figure S5C). No effect on aboveground C or N content was observed for nutrient content (Figures S5A,B). Belowground C content significantly increased 14% over the entire range of nutrient treatments at 25°C (*P* < 0.01; Figure S5D), while belowground N and P content were not affected by nutrient treatment (Table 1).

#### Abiotic conditions

**Figure 3:**
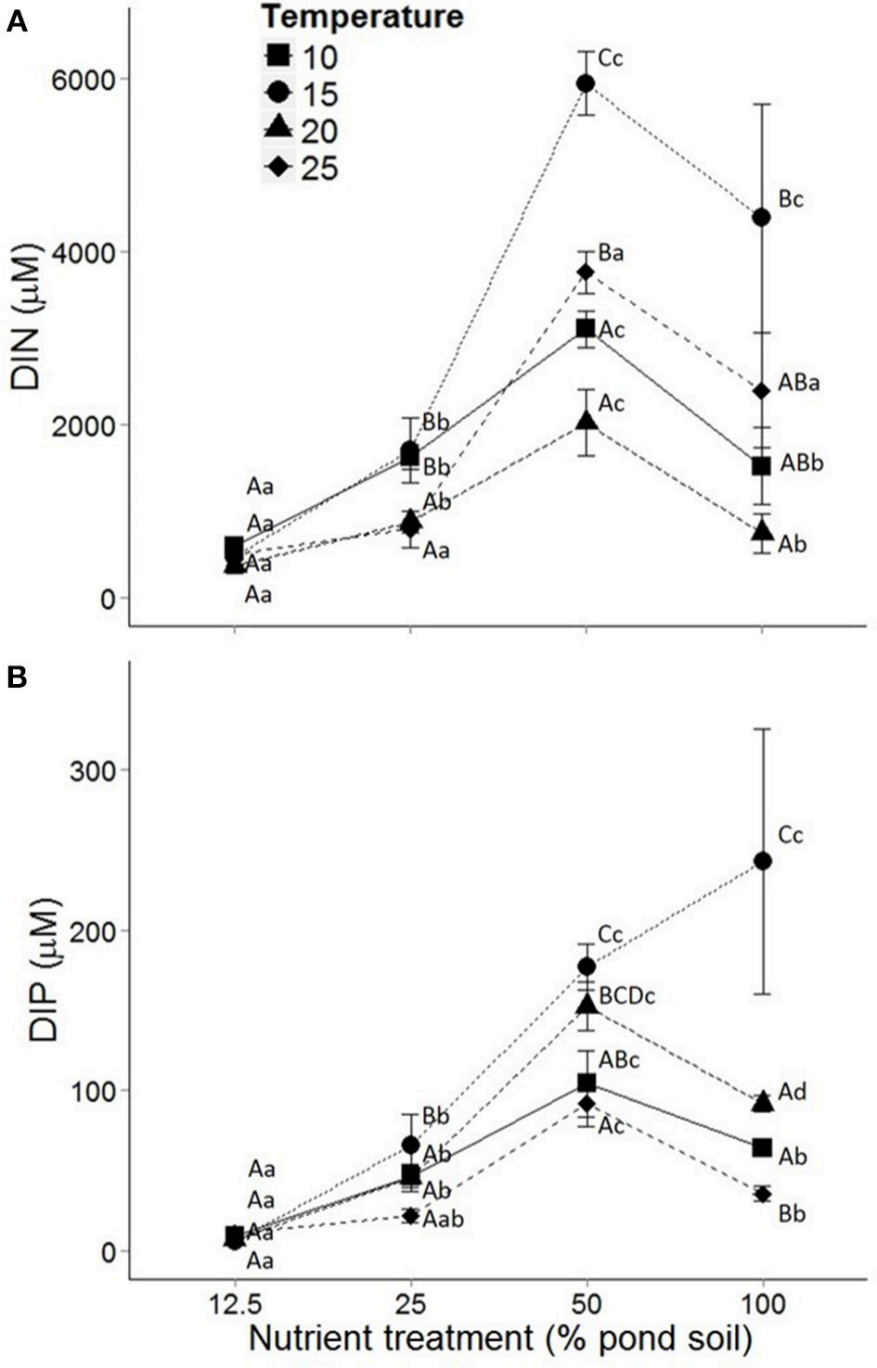
**Dissolved nutrient concentrations in the pore water in response to sediment nutrient content**, with dissolved inorganic nitrogen (DIN) **(A)** and dissolved inorganic phosphorus (DIP) **(B)** at the end of the experiment. Temperature treatments include 10 (■), 15 (•), 20 (▴) and 25 (♦)°C. Dots represent means and error bars standard error of the mean. Capital and lower case letters indicate *post-hoc* differences between temperature and nutrient treatments, respectively.

Temperature affected dissolved nutrient concentrations in the pore water, and this effect interacted with nutrient treatment (Table 1). Temperature effects on pore water DIN concentrations were strongest at intermediate sediment nutrient content (50%), where values significantly doubled from 10 to 15°C, and decreased at higher temperatures (*P* < 0.01, Tukey *post-hoc* comparison; Figure 3A). Pore water DIP concentrations were significantly higher at 15°C than other temperature treatments in the highest nutrient treatment (*P* < 0.01; Figure 3B). This response was less pronounced in other nutrient treatments. Similarly to temperature, sediment nutrient content affected DIN and DIP concentrations in the pore water. Pore water DIN and DIP increased 7- and 16-fold, respectively, from the 12.5 to 50% nutrient treatment irrespective of temperature (*P* < 0.01; Figures 3A,B).

### Meta-analysis

#### Effects of elevated temperature on carbon:nutrient stoichiometry

**Figure 4:**
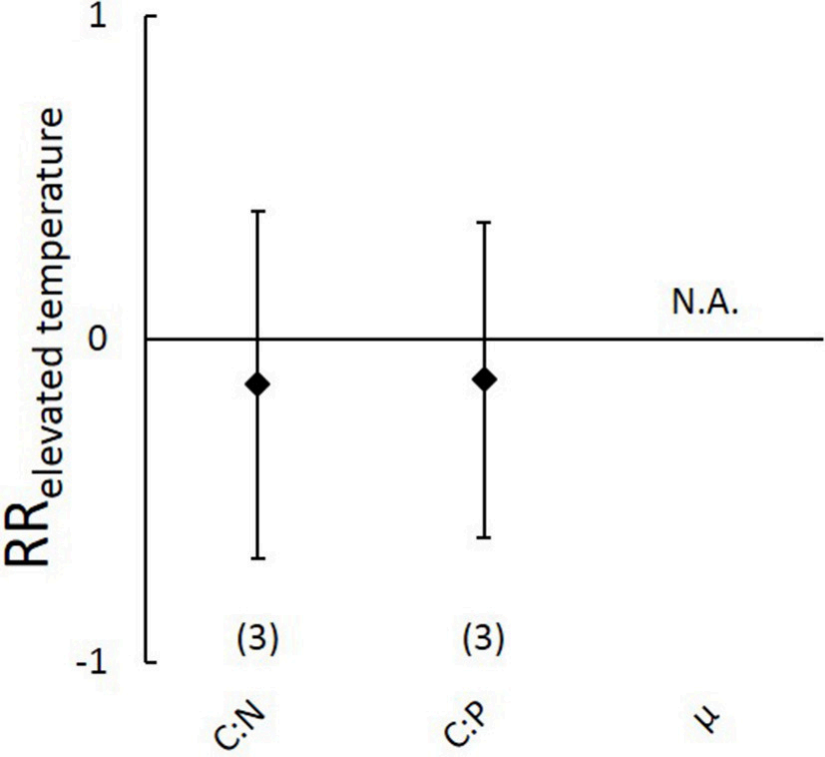
**Natural-log response ratios of aboveground carbon:nutrient stoichiometry and plant growth rates (μ) to 3-6 degrees elevated temperature from the meta-analysis on submerged aquatic plants**. Values represent means, error bars 95% confidence intervals and sample size is indicated between brackets. No response ratios were significantly different from zero. N.A., indicates that data were not available.

No significant effects of elevated temperature were observed on aboveground C:N and C:P ratios (Figure 4), nor on aboveground C, N and P contents (Figure S6A) or belowground N and P contents (Figure S6B). Sample sizes were too low to analyze effects of elevated temperature on aboveground growth rates (*n* = 0), on belowground C:N and C:P ratios and C content (*n* = 1) or on potential differences between marine and freshwater ecosystems (*n* = 1 and 2 respectively).

#### Effects of nutrient addition on carbon:nutrient stoichiometry

**Figure 5:**
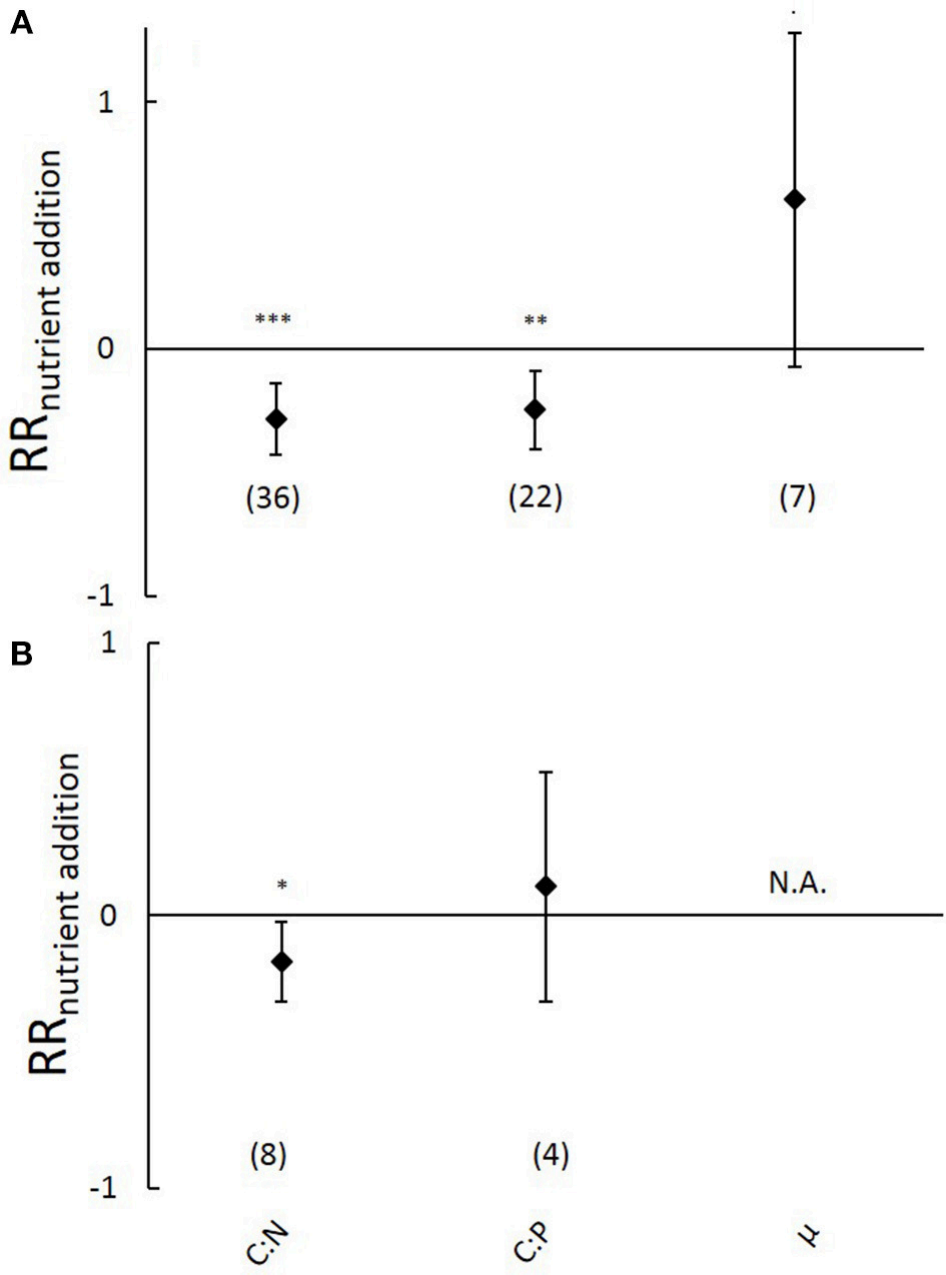
**Natural-log response ratios of carbon:nutrient stoichiometry and plant growth rates (μ) to nutrient (nitrogen and phosphorus) addition in (A)** above- and **(B)** belowground biomass of submerged aquatic plants. Values represent means, error bars 95% confidence intervals and sample size is indicated between brackets. Response ratios significantly different from zero are indicated as follows: ^***^P < 0.001, ^**^P < 0.01, ^*^P < 0.05 and ^·^*P* < 0.10. N.A., indicates that data were not available.

Nutrient addition significantly decreased aboveground carbon:nutrient ratios, with 24.7 and 21.9% for C:N and C:P ratios, respectively (Figure 5A). This decrease in aboveground carbon:nutrient ratios was accompanied by a 23.5% increase in aboveground N content and a tendency for increased P content (with 20.6%, *P* = 0.06), while C content remained unaffected (Figure S6C). Furthermore, aboveground growth rates tended to increase 83.1% with nutrient addition, but this effect was not significant (*P* = 0.08, Figure 5A). Similar to aboveground responses, belowground C:N ratio also declined 15.6% with nutrient addition, while no effect on belowground C:P ratios was observed (Figure 5B). This decline in C:N ratio was accompanied by an 18.2% increase in belowground N content (Figure S6D).

**Figure 6:**
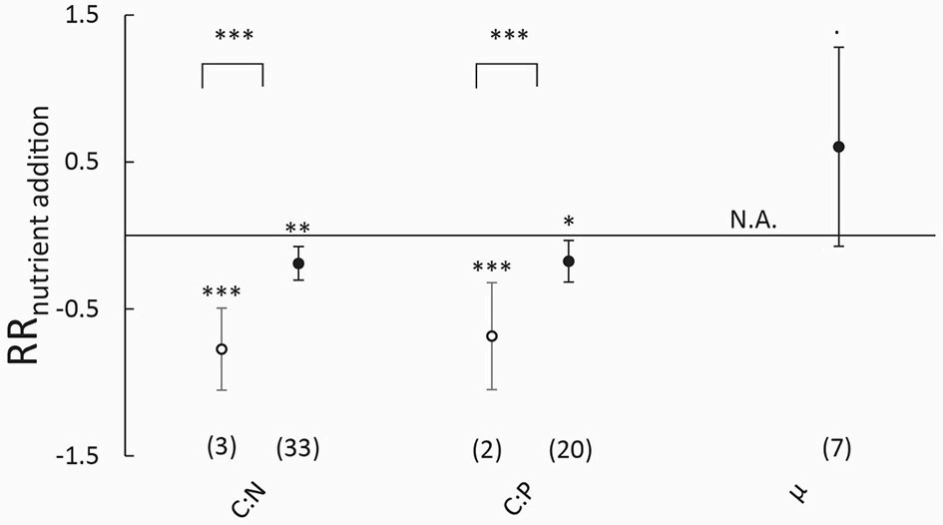
**Natural-log response ratios of aboveground carbon:nutrient stoichiometry and growth rates (μ) to nutrient (nitrogen and phosphorus) addition in freshwater (open circles) and marine (closed circles) submerged aquatic plants**. Values represent means, error bars 95% confidence intervals and sample size is indicated between brackets. Significance levels are indicated as follows: ^***^P < 0.001, ^**^P < 0.01, ^*^P < 0.05 and ^·^*P* < 0.10. N.A., indicates that data were not available.

Aboveground carbon:nutrient stoichiometry of marine and freshwater plants responded qualitatively similar to nutrient addition, though the number of studies in the latter group was far lower (Figure 6). Quantitatively, responses in C:N and C:P were stronger for freshwater compared to marine plants (*P* < 0.001). Sample sizes were too low to analyze differences in aboveground growth rates between freshwater and marine plants (*n* = 0 for freshwater plants).

## Discussion

To address the impacts of temperature and nutrient availability on the growth and carbon:nutrient stoichiometry of aquatic plants, we performed a microcosm experiment and a meta-analysis. In line with our first hypothesis, elevated temperatures led to higher growth rates and standing stock biomass of the freshwater plant *Elodea nuttallii*, with an optimal growth at 15°C. In contrast to the biomass-dilution effect (second hypothesis), aboveground C:N ratios were negatively affected by temperature, and this effect interacted with nutrient treatment. Aboveground C:P ratios of E. *nuttallii* were lower with higher sediment nutrient content, in line with our third hypothesis. The observed decrease in aboveground C:P ratio coincided with an increase in P content, confirming our fourth hypothesis. However, in contrast to our third and fourth hypotheses, belowground C:N and C:P ratios as well as belowground C content increased with higher sediment nutrient content.

In the meta-analysis, elevated temperature did not lead to enhanced growth rates or increased carbon:nutrient ratios of submerged aquatic plants in general, in contrast to our first and second hypotheses. However, it should be noted that overall sample sizes were very low (*n* = 3), which may (partly) explain the lack of effect. In line with our first hypothesis, nutrient (e.g., nitrogen and phosphorus) addition tended to increase plant growth rates, though this effect was not significant. Nutrient addition led to decreased C:N and C:P ratios and increased N content, in agreement with the third and fourth hypotheses. The carbon:nutrient ratio declined in both marine and freshwater plants upon nutrient addition, although the absolute level of the response was stronger in freshwater systems.

### Effects of temperature on plant carbon:nutrient stoichiometry

Aboveground C:N ratio of *E. nuttallii* decreased moderately with increasing temperatures in our experiment. This is in direct contrast with the hypothesis of enhanced nutrient-use efficiency with elevated temperatures (2), which would lead to increased carbon:nutrient ratios [as is observed for other aquatic plants such as *Zostera marina* (Kaldy, 2014)]. The decrease in C:N ratios was most pronounced between 10 and 25°C, even though the aboveground biomass did not differ between those temperatures. Thus, temperature does not seem to indirectly affect C:N ratios through changes in biomass. Accompanied by the decreased C:N ratios in our *Elodea* experiment with higher temperatures, N contents tended to be higher as well, but this effect was not significant. Increased N content over similar temperature ranges has been documented for *E. canadensis* (Ventura et al., 2008) and *Ruppia drepanensis* (Santamaria and Hootsmans, 1998) and could indicate resource allocation to nitrogen-rich compounds such as chlorophyll-a (Santamaria and Hootsmans, 1998). Furthermore, elevated temperature can increase nitrogen availability in the sediment pore water through enhanced nitrogen mobilization (Alsterberg et al., 2012), thereby indirectly leading to higher nitrogen availability for plant growth. In our experiment, the temperature treatments with highest aboveground biomass of *E. nuttallii* (e.g., 15 and 20°C) varied considerably in their pore water nitrogen availability, indicating that those are not directly related. Furthermore, as the temperature effect on C:N ratios was most pronounced at intermediate sediment nutrient content (as indicated by the temperature × nutrient interaction term), these results indicate that stoichiometric responses of plants to changes in temperature may be directly and indirectly altered by nutrient availability.

In our meta-analysis, we observed no overall effect of an 3–6°C elevated temperature on carbon:nutrient ratios of submerged aquatic plants, which contradicts findings in other groups of primary producers, such as phytoplankton (Toseland et al., 2013; De Senerpont Domis et al., 2014) and terrestrial plants (An et al., 2005). The number of studies in our analysis was rather low (*n* = 3) and included a positive (Zhang et al., 2016), negative (Ventura et al., 2008), and neutral (Touchette et al., 2003) response. The different directions of responses indicate that effects of temperature on the carbon:nutrient stoichiometry of aquatic plants are not necessarily linked to the temperature increments they are exposed to. Thus, it may indicate species-specific responses or possibly even a phylogenetic relationship considering the similar response of *E. nuttallii* in our experiment and *E. canadensis* (Ventura et al., 2008), which both have an optimal growth temperature of around 15°C (Olesen and Madsen, 2000). However, due to the limited sample size of each species (*n* = 1), we currently cannot distinguish between species-specific and study-specific responses (such as experimental set-up and environmental conditions) of carbon:nutrient stoichiometry in our analysis.

### Effects of nutrient addition on carbon:nutrient tissue stoichiometry

Aboveground C:P ratios decreased about 4-fold with increasing sediment nutrient content in the *Elodea* experiment, confirming our third hypothesis. However, in contrast to this hypothesis, belowground C:P ratios of *E. nuttallii* and carbon content rather increased with sediment nutrient availability. Higher belowground carbon content can indicate thicker cell walls and thicker roots. Possibly, with sufficient nutrient availability, *Elodea* may shift from investment in root structures for nutrient uptake to thicker roots for anchorage in the sediment (Sand-Jensen and Madsen, 1991). In the meta-analysis, nutrient addition led to a 25% and 22% decrease in aboveground C:N and C:P ratios of submerged aquatic plants, consistent with the results from the *Elodea* experiment. While some variability in response can be detected at the species level, responses are consistently either absent or negative (Figure S7). Similar to the aboveground responses, nutrient addition led to a 16% decrease in belowground C:N ratios. These decreases in above- and belowground carbon:nutrient ratios were accompanied by increased tissue N and P contents and demonstrate the flexibility in carbon:nutrient stoichiometry of aquatic plants under fluctuating nutrient availability (Sardans et al., 2012).

Increased plant nutrient content as observed in our meta-analysis and *Elodea* experiment may have resulted from excess or luxurious uptake of nutrients (Millard, 1988), as terrestrial plants can store excess P in cell vacuoles (Bieleski, 1973) and N in specialized storage organs (Aerts and Chapin, 2000). Similar to our results, meta-analytic studies on terrestrial plants observed elevated foliar N and P contents in response to nutrient addition (Yuan and Chen, 2015) and a decrease in C:N in photosynthetic tissues to N addition (Sardans et al., 2012). Combined with our results, this indicates that these effects are not ecosystem specific, but can be seen as a general qualitative response of primary producers to nutrient addition.

Our analysis indeed indicated qualitatively similar responses to nutrient addition in both marine and freshwater submerged plants, though the responses were stronger in the latter group. Sample sizes for freshwater plants were far lower than for marine plants, highlighting the potential for freshwater research to learn from physiological studies on marine plants. Mean C:N ratios of freshwater plants are lower than marine plants (Bakker et al., 2016) and could result from higher levels of fertilization as nutrient levels in freshwater are generally considered higher than in marine systems (Smith et al., 1999). However, as these ecosystems differ greatly in retention time, sediment characteristics and osmotic stress from salinity (Short et al., 2016), caution must be taken when interpreting these differences.

### Possible implications for carbon cycling and food-web dynamics

Changes in plant carbon:nutrient stoichiometry in aquatic systems can have consequences for carbon cycling. In our meta-analysis, nutrient addition tended to increase plant growth rates, with positive (Murray et al., 1992; Udy et al., 1999; Peralta et al., 2003) and neutral (Erftemeijer et al., 1994; Holzer and McGlathery, 2016) responses reported. Thus, carbon sequestration in the form of plant standing stock biomass can be enhanced by nutrient addition (Armitage and Fourqurean, 2016). Furthermore, changes in carbon:nutrient stoichiometry can have consequences for the energy transfer to higher trophic levels as elevated nutrient content in aquatic plants can lead to increased herbivore grazing rates (Bakker and Nolet, 2014) and subsequent reduction in standing-stock biomass (van Altena et al., 2016). This may counteract positive effects of fertilization on plant growth rates and carbon sequestration. Furthermore, eutrophic conditions can enhance plant litter quality (Emsens et al., 2016) and plants with lower carbon:nutrient ratios decompose faster than those with higher ratios (Wang et al., 2017), indicating an accelerated release of sequestered carbon and nutrients. We therefore hypothesize that eutrophication can affect carbon stocks in submerged aquatic vegetation, through changes in their nutritional quality (e.g., reduced carbon:nutrient stoichiometry) and subsequent effects on grazing and decomposition. Given the current knowledge about the effects of temperature on carbon:nutrient stoichiometry of aquatic plants presented in this study, we cannot draw any general conclusions on the effect of global warming on aquatic carbon cycling. However, our results suggest species-specific responses, which indicates that given the community composition in an ecosystem, effects may be substantial. Our current analysis focuses on individual plant responses and their stoichiometric flexibility. On a community level, interspecific variability can drive changes in C:N:P stoichiometry (Frost and Hicks, 2012), with consequences for community composition under elevated nutrient availability and temperature. For instance, elevated temperature can shift aquatic plant community composition toward floating vegetation (Netten et al., 2010), while nutrient addition can lead to a decline in overall plant abundance at the expense of algae (Scheffer et al., 1993; Short and Neckles, 1999). Therefore, hypotheses on an ecosystem level should also take these changes into account.

## Conclusion

We conclude that nutrient (e.g., nitrogen and phosphorus) addition decreases carbon:nutrient stoichiometry in submerged aquatic plants, while no consistent effects of elevated temperature on these ratios were observed. The latter could be an effect of low sample size or could indicate species-specific responses in carbon:nutrient stoichiometry to global warming, which is an interesting avenue for future research. Furthermore, our experiment shows that the impact of temperature on aquatic plant stoichiometry depends on the availability of nutrients for plant growth, which is seldom taken into account. The impact of temperature may thus be modified by nutrient availability. The observed decline in carbon:nutrient stoichiometry of aquatic plants in response to nutrient addition can stimulate the further energy transfer to herbivores and decomposers, leading to reduced carbon stocks. With ongoing global warming, the knowledge gap of temperature effects on carbon:nutrient stoichiometry of submerged aquatic plants is in urgent need for further investigation.

## Author contributions

MV, EB, and EvDo conceived and designed the experiments. EvDe performed the experiments. MV, EvDe, and PZ analyzed the data. MV and EB wrote the manuscript; all other authors provided editorial contributions.

## Funding

The work of MV is funded by the Gieskes-Strijbis Foundation and the work of PZ by the China Scholarship Council (CSC).

### Conflict of interest statement

The authors declare that the research was conducted in the absence of any commercial or financial relationships that could be construed as a potential conflict of interest.

## References

[B1] Adrian R. O'Reilly C. M. Zagarese H. Baines S. B. Hessen D. O. Keller W.. (2009). Lakes as sentinels of climate change. Limnol. Oceanogr. 54, 2283–2297. 10.4319/lo.2009.54.6_part_2.2283 20396409 PMC2854826

[B2] Aerts R. Chapin F. S. III. (2000). The mineral nutrition of wild plants revisited: a re-evaluation of processes and patterns. Adv. Ecol. Res. 30, 1–67. 10.1016/S0065-2504(08)60016-1

[B3] Alsterberg C. Sundbäck K. Hulth S. (2012). Functioning of a shallow-water sediment system during experimental warming and nutrient enrichment. PLoS ONE 7:e51503. 10.1371/journal.pone.0051503 23240032 PMC3519877

[B4] An Y. Wan S. Zhou X. Subedar A. A. Wallace L. L. Luo Y. (2005). Plant nitrogen concentration, use efficiency, and contents in a tallgrass prairie ecosystem under experimental warming. Glob. Chang. Biol. 11, 1733–1744. 10.1111/j.1365-2486.2005.01030.x

[B5] Armitage A. R. Fourqurean J. W. (2016). Carbon storage in seagrass soils: long-term nutrient history exceeds the effects of near-term nutrient enrichment. Biogeosciences 13, 313–321. 10.5194/bg-13-313-2016

[B6] Bakker E. S. Nolet B. A. (2014). Experimental evidence for enhanced top-down control of freshwater macrophytes with nutrient enrichment. Oecologia 176, 825–836. 10.1007/s00442-014-3047-y 25194349 PMC4207960

[B7] Bakker E. S. Wood K. A. Pagès J. F. Veen G. F. Christianen M. J. A. Santamaría L. (2016). Herbivory on freshwater and marine macrophytes: a review and perspective. Aquat. Bot. 135, 18–36. 10.1016/j.aquabot.2016.04.008

[B8] Benjamini Y. Hochberg Y. (1995). Controlling the false discovery rate: a practical and powerful approach to multiple testing. J. R. Stat. Soc. Ser. B 57, 289–300.

[B9] Bieleski R. L. (1973). Phosphate pools, phosphate transport, and phosphate availability. Annu. Rev. Plant Physiol. Plant Mol. Biol. 24, 225–252. 10.1146/annurev.pp.24.060173.001301

[B10] Bloom A. J. Chapin F. S. Mooney H. A. (1985). Resource limitation in plants - an economic analogy. Annu. Rev. Ecol. Syst. 16, 363–392. 10.1146/annurev.es.16.110185.002051

[B11] Bornette G. Puijalon S. (2011). Response of aquatic plants to abiotic factors: a review. Aquat. Sci. 73, 1–14. 10.1007/s00027-010-0162-7

[B12] Burkholder J. M. Tomasko D. A. Touchette B. W. (2007). Seagrasses and eutrophication. J. Exp. Mar. Biol. Ecol. 350, 46–72. 10.1016/j.jembe.2007.06.024

[B13] Carpenter S. R. Caraco N. F. Correll D. L. Howarth R. W. Sharpley A. N. Smith V. H. (1998). Nonpoint pollution of surface waters with phosphorus and nitrogen. Ecol. Appl. 8, 559–568. 10.1890/1051-0761(1998)008[0559:NPOSWW]2.0.CO;2

[B14] Collaboration for Environmental Evidence (2013). Guidelines for Systematic Review and Evidence Synthesis in Environmental Management. Version 4.2. Environmental Evidence: Available online at: www.environmentalevidence.org/Documents/Guidelines/Guidelines4.2.pdf

[B15] Cook C. D. K. Urmi-König K. (1985). A revision of the genus Elodea (Hydrocharitaceae). Aquat. Bot. 21, 111–156. 10.1016/0304-3770(85)90084-1

[B16] De Senerpont Domis L. N. Van de Waal D. B. Helmsing N. R. Donk E. Mooij W. M. (2014). Community stoichiometry in a changing world: combined effects of warming and eutrophication on phytoplankton dynamics. Ecology 95, 1485–1495. 10.1890/13-1251.1 25039214

[B17] Dorenbosch M. Bakker E. S. (2011). Herbivory in omnivorous fishes: effect of plant secondary metabolites and prey stoichiometry. Freshw. Biol. 56, 1783–1797. 10.1111/j.1365-2427.2011.02618.x

[B18] Emsens W. J. Aggenbach C. J. Grootjans A. P. Nfor E. E. Schoelynck J. Struyf E.. (2016). Eutrophication triggers contrasting multilevel feedbacks on litter accumulation and decomposition in fens. Ecology 97, 2680–2690. 10.1002/ecy.1482 27859133

[B19] Erftemeijer P. L. A. Stapel J. Smekens M. J. E. Drossaert W. M. E. (1994). The limited effect of *in-situ* phosphorus and nitrogen additions to seagrass beds on carbonate and terrigenous sediments in South Sulawesi, Indonesia. J. Exp. Mar. Biol. Ecol. 182, 123–140. 10.1016/0022-0981(94)90215-1

[B20] Frost P. C. Hicks A. L. (2012). Human shoreline development and the nutrient stoichiometry of aquatic plant communities in Canadian Shield lakes. Can. J. Fish. Aquat. Sci. 69, 1642–1650. 10.1139/f2012-080

[B21] Heithaus M. R. Alcoverro T. Arthur R. Burkholder D. A. Coates K. A. Christianen M. J. A. (2014). Seagrasses in the age of sea turtle conservation and shark overfishing. Front. Mar. Sci. 1:28 10.3389/fmars.2014.00028

[B22] Holzer K. K. McGlathery K. J. (2016). Cultivation grazing response in seagrass may depend on phosphorus availability. Mar. Biol. 163:88 10.1007/s00227-016-2855-5

[B23] Hothorn T. Bretz F. Westfall P. (2008). Simultaneous inference in general parametric models. Biometrical J. 50, 346–363. 10.1002/bimj.200810425 18481363

[B24] IPCC (2014). Climate Change 2014: Synthesis Report. Contribution of Working Groups I, II and III to the Fifth Assessment Report of the Intergovernmental Panel on Climate Change. Geneva: IPCC

[B25] Kaldy J. E. (2014). Effect of temperature and nutrient manipulations on eelgrass *Zostera marina* L. from the Pacific Northwest, USA. J. Exp. Mar. Biol. Ecol. 453, 108–115. 10.1016/j.jembe.2013.12.020

[B26] Lajeunesse M. J. (2015). Bias and correction for the log response ratio in ecological meta-analysis. Ecology 96, 2056–2063. 10.1890/14-2402.1 26405731

[B27] Millard P. (1988). The accumulation and storage of nitrogen by herbaceous plants. Plant Cell Environ. 11, 1–8. 10.1111/j.1365-3040.1988.tb01769.x

[B28] Mooij W. M. De Senerpont Domis L. N. Hülsmann S. (2008). The impact of climate warming on water temperature, timing of hatching and young-of-the-year growth of fish in shallow lakes in the Netherlands. J. Sea Res. 60, 32–43. 10.1016/j.seares.2008.03.002

[B29] Murray L. Dennison W. C. Kemp W. M. (1992). Nitrogen versus phosphorus limitation for growth of an estuarine population of eelgrass (*Zostera marina* L.). Aquat. Bot. 44, 83–100. 10.1016/0304-3770(92)90083-U

[B30] Netten J. J. C. Arts G. H. P. Gylstra R. Nes E. H. Scheffer M. Roijackers R. M. M. (2010). Effect of temperature and nutrients on the competition between free-floating Salvinia natans and submerged *Elodea nuttallii* in mesocosms. Fundam. Appl. Limnol. 177, 125–132. 10.1127/1863-9135/2010/0177-0125

[B31] Olesen B. Madsen T. V. (2000). Growth and physiological acclimation to temperature and inorganic carbon availability by two submerged aquatic macrophyte species, *Callitriche cophocarpa* and *Elodea canadensis*. Funct. Ecol. 14, 252–260. 10.1046/j.1365-2435.2000.00412.x

[B32] Olsen Y. S. Valiela I. (2010). Effect of sediment nutrient enrichment and grazing on turtle grass *Thalassia testudinum* in Jobos Bay, Puerto Rico. Estuaries Coasts 33, 769–783. 10.1007/s12237-009-9256-7

[B33] Peralta G. Bouma T. J. van Soelen J. Perez-Llorens J. L. Hernandez I. (2003). On the use of sediment fertilization for seagrass restoration: a mesocosm study on *Zostera marina* L. Aquat. Bot. 75, 95–110. 10.1016/S0304-3770(02)00168-7

[B34] R Core Team (2015). R: A Language and Environment for Statistical Computing. Vienna: R Foundation for statistical computing Available online at: https://www.R-project.org/

[B35] Sand-Jensen K. Madsen T. V. (1991). Minimum light requirements of submerged freshwater macrophytes in laboratory growth experiments. J. Ecol. 79, 749–764. 10.2307/2260665

[B36] Santamaria L. Hootsmans M. J. M. (1998). The effect of temperature on the photosynthesis, growth and reproduction of a Mediterranean submerged macrophyte, Ruppia drepanensis. Aquat. Bot. 60, 169–188. 10.1016/S0304-3770(97)00050-8

[B37] Sardans J. Rivas-Ubach A. Penuelas J. (2012). The C:N:P stoichiometry of organisms and ecosystems in a changing world: a review and perspectives. Perspect. Plant Ecol. Evol. Syst. 14, 33–47. 10.1016/j.ppees.2011.08.002

[B38] Scheffer M. Hosper S. H. Meijer M. L. Moss B. Jeppesen E. (1993). Alternative equilibria in shallow lakes. Trends Ecol. Evol. (Amst). 8, 275–279. 10.1016/0169-5347(93)90254-M 21236168

[B39] Short F. T. Kosten S. Morgan P. A. Malone S. Moore G. E. (2016). Impacts of climate change on submerged and emergent wetland plants. Aquat. Bot. 135, 3–17. 10.1016/j.aquabot.2016.06.006

[B40] Short F. T. Neckles H. A. (1999). The effects of global climate change on seagrasses. Aquat. Bot. 63, 169–196. 10.1016/S0304-3770(98)00117-X

[B41] Smith V. H. Tilman G. D. Nekola J. C. (1999). Eutrophication: impacts of excess nutrient inputs on freshwater, marine, and terrestrial ecosystems. Environ. Pollut. 100, 179–196. 10.1016/S0269-7491(99)00091-3 15093117

[B42] Steffen W. Richardson K. Rockström J. Cornell S. E. Fetzer I. Bennett E. M.. (2015). Planetary boundaries: guiding human development on a changing planet. Science 347:1259855. 10.1126/science.1259855 25592418

[B43] Sterner R. W. Elser J. J. (2002). Ecological Stoichiometry: the Biology of Elements from Molecules to the Biosphere. Princeton, NJ: Princeton University Press.

[B44] Taylor W. D. Carey J. H. Lean D. R. S. McQueen D. J. (1991). Organochlorine concentrations in the plankton of lakes in southern Ontario and their relationship to plankton biomass. Can. J. Fish. Aquat. Sci. 48, 1960–1966. 10.1139/f91-233

[B45] Tilman D. Fargione J. Wolff B. D'Antonio C. Dobson A. Howarth R.. (2001). Forecasting agriculturally driven global environmental change. Science 292, 281–284. 10.1126/science.1057544 11303102

[B46] Toseland A. Daines S. J. Clark J. R. Kirkham A. Strauss J. Uhlig C. (2013). The impact of temperature on marine phytoplankton resource allocation and metabolism. Nat. Clim. Chang. 3, 979–984. 10.1038/nclimate1989

[B47] Touchette B. W. Burkholder J. M. Glasgow H. B. (2003). Variations in eelgrass (*Zostera marina* L.) morphology and internal nutrient composition as influenced by increased temperature and water column nitrate. Estuaries 26, 142–155. 10.1007/BF02691701

[B48] Udy J. W. Dennison W. C. Long W. J. L. McKenzie L. J. (1999). Responses of seagrass to nutrients in the Great Barrier Reef, Australia. Mar. Ecol. Prog. Ser. 185, 257–271. 10.3354/meps185257

[B49] van Altena C. Bakker E. S. Kuiper J. J. Mooij W. M. (2016). The impact of bird herbivory on macrophytes and the resilience of the clear-water state in shallow lakes: a model study. Hydrobiologia 777, 197–207. 10.1007/s10750-016-2779-6

[B50] van Dam H. (2009). Evaluatie Basismeetnet Waterkwaliteit Hollands Noorderkwartier: Trendanalyse Hydrobiology, Temperatuur en Waterchemie 1982–2007. Edam: Hoogheemraadschap Hollands Noorderkwartier.

[B51] Ventura M. Liboriussen L. Lauridsen T. M. SØndergaard M. SØndergaard Jeppesen E. (2008). Effects of increased temperature and nutrient enrichment on the stoichiometry of primary producers and consumers in temperate shallow lakes. Freshw. Biol. 53, 1434–1452. 10.1111/j.1365-2427.2008.01975.x

[B52] Vermaat J. E. Hootsmans M. J. M. (1994). Growth of *Potamogeton pectinatus* L. in a temperature-light gradient, in Lake Veluwe, a Macrophyte-Dominated System under Eutrophication Stress, eds van Vierssen W. Hootsmans M. Vermaat J. (Dordrecht: Springer Netherlands), 40–61.

[B53] Viechtbauer W. (2010). Conducting meta-analyses in R with the metafor package. J. Stat. Softw. 36, 1–48. 10.18637/jss.v036.i03

[B54] Vitousek P. M. Mooney H. A. Lubchenco J. Melillo J. M. (1997). Human domination of earth's ecosystems. Science 277, 494–499. 10.1126/science.277.5325.494

[B55] Wang M. Hao T. Deng X. W. Wang Z. X. Cai Z. H. Li Z. Q. (2017). Effects of sediment-borne nutrient and litter quality on macrophyte decomposition and nutrient release. Hydrobiologia 787, 205–215. 10.1007/s10750-016-2961-x

[B56] Yuan Z. Y. Chen H. Y. H. (2015). Negative effects of fertilization on plant nutrient resorption. Ecology 96, 373–380. 10.1890/14-0140.1 26240859

[B57] Zhang P. Y. Bakker E. S. Zhang M. Xu J. (2016). Effects of warming on Potamogeton crispus growth and tissue stoichiometry in the growing season. Aquat. Bot. 128, 13–17. 10.1016/j.aquabot.2015.08.004

