# Supplementary material for: Impact of Temperature and Nutrients on Carbon: Nutrient Tissue Stoichiometry of Submerged Aquatic Plants: An Experiment and Meta-Analysis
Source: Front Plant Sci. 2017 May 4;8:655. doi: 10.3389/fpls.2017.00655 (PMC5416745; doi:10.3389/fpls.2017.00655)
Supplement: Supplementary file 1 [file DataSheet1.DOCX]

Supplementary material S1 **Abiotic conditions**

**Methods**

During the final harvest, PH was measured in each microcosm with a SenTix 41 pH electrode (WTW GmbH, Weilheim, Germany). From the middle of each microcosm, water samples were taken for sestonic chlorophyll-a concentrations (as a proxy for phytoplankton biomass), alkalinity and dissolved nutrient concentrations. Chlorophyll-a concentrations were approximated by chlorophyll-a fluorescence, which was measured on a Phyto-PAM with an Optical Unit ED-101US/MP (Heinz Walz GmbH, Effeltrich, Germany), using a 0.2 µm filtered water sample for background correction. Water samples for alkalinity and dissolved nutrient concentrations were taken from the middle of the microcosm, filtered over prewashed GF/F filters (Whatman, Maidstone, U.K.) and stored at 4°C and -20°C, respectively, until further analysis. Alkalinity was determined at room temperature by titration to pH 4.2 with 0.1 N HCl on a Tim840 Titration manager (Hach Lange, Loveland, Colorado, U.S.). Concentrations of dissolved nutrients (PO_4_^3-^, NO_2_^-^, NO_3_^-^ and NH_4_^+^) of thawed samples were determined on a QuAAtro39 Auto-Analyzer **(**SEAL Analytical Ltd**.,** Southampton, U.K.).

**Results**

Table S1. Summary of generalized linear model analysis of the *Elodea* experiment, describing the effect of temperature, nutrient treatment and their interaction on seston chlorophyll-a concentrations, pH and alkalinity. Significant results are indicated in bold, with ***:P<0.001, **:P<0.01 and *:P<0.05.

|  |  | Chi-square values | | |
| --- | --- | --- | --- | --- |
| **Variable** | Unit | Temperature | Nutrients | Temperature × Nutrients |
| Chlorophyll-a | µg/L | **9.8*** | **39.5***** | 7.3 |
| pH |  | **52..3***** | 3.5 | **17.5*** |
| Alkalinity | meq/L | **23.6***** | **15.1**** | 14.5 |
| Water column DIN | µM | **10.0*** | **206.8***** | **18.1*** |
| Water column DIP | µM | **138.0***** | **23.4***** | **252.6***** |


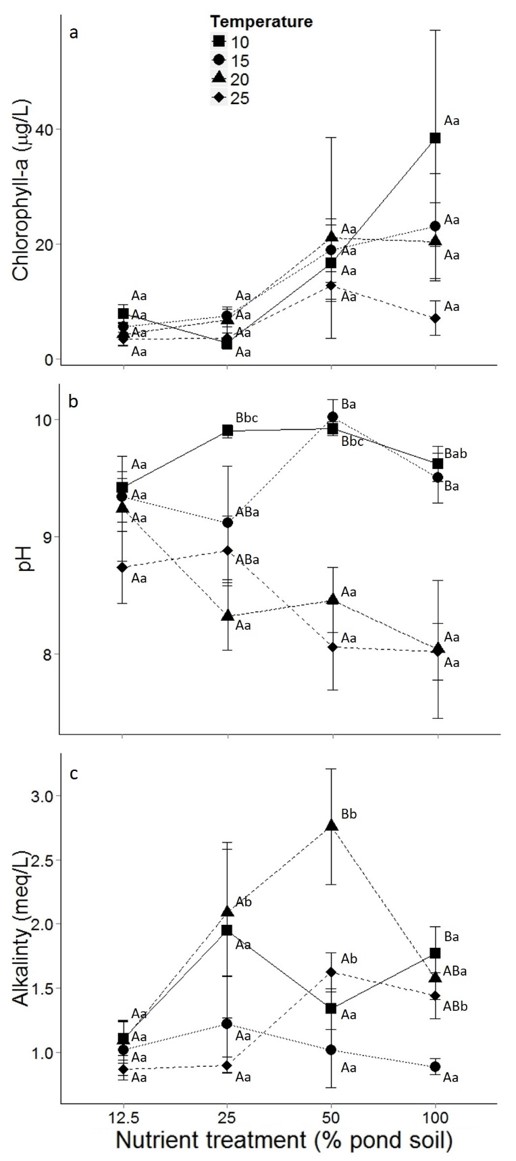


Fig. S1.1. Environmental conditions in the *Elodea* experiment in response to sediment nutrient content, with (A) sestonic chlorophyll-a concentrations, (B) pH and (C) alkalinity at the end of the experiment. Temperature treatments include 10 (■), 15 (●), 20 (▲) and 25 (♦) °C. Dots represent means and error bars standard error of the mean. Capital and lower case letters indicate post-hoc differences between temperature and nutrient treatments, respectively.


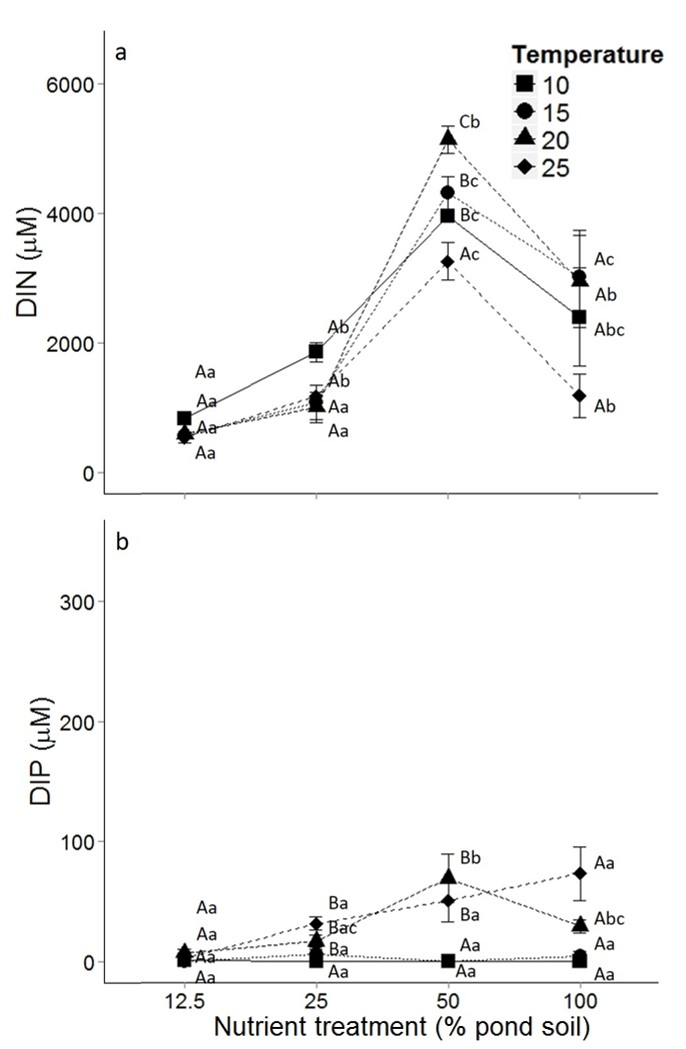


Fig. S1.2. Dissolved nutrient concentrations in the water column in response to nutrient treatment, with dissolved organic nitrogen (DIN) (a) and dissolved organic phosphorus (DIP) (b) at the end of the experiment. Temperature treatments include 10 (■), 15 (●), 20 (▲) and 25 (♦) °C. Dots represent means and error bars standard error of the mean. Capital and lower case letters indicate post-hoc differences between temperature and nutrient treatments, respectively.
