# Supplementary material for: Impact of Temperature and Nutrients on Carbon: Nutrient Tissue Stoichiometry of Submerged Aquatic Plants: An Experiment and Meta-Analysis
Source: Front Plant Sci. 2017 May 4;8:655. doi: 10.3389/fpls.2017.00655 (PMC5416745; doi:10.3389/fpls.2017.00655)
Supplement: Supplementary file 2 [file DataSheet2.DOCX]

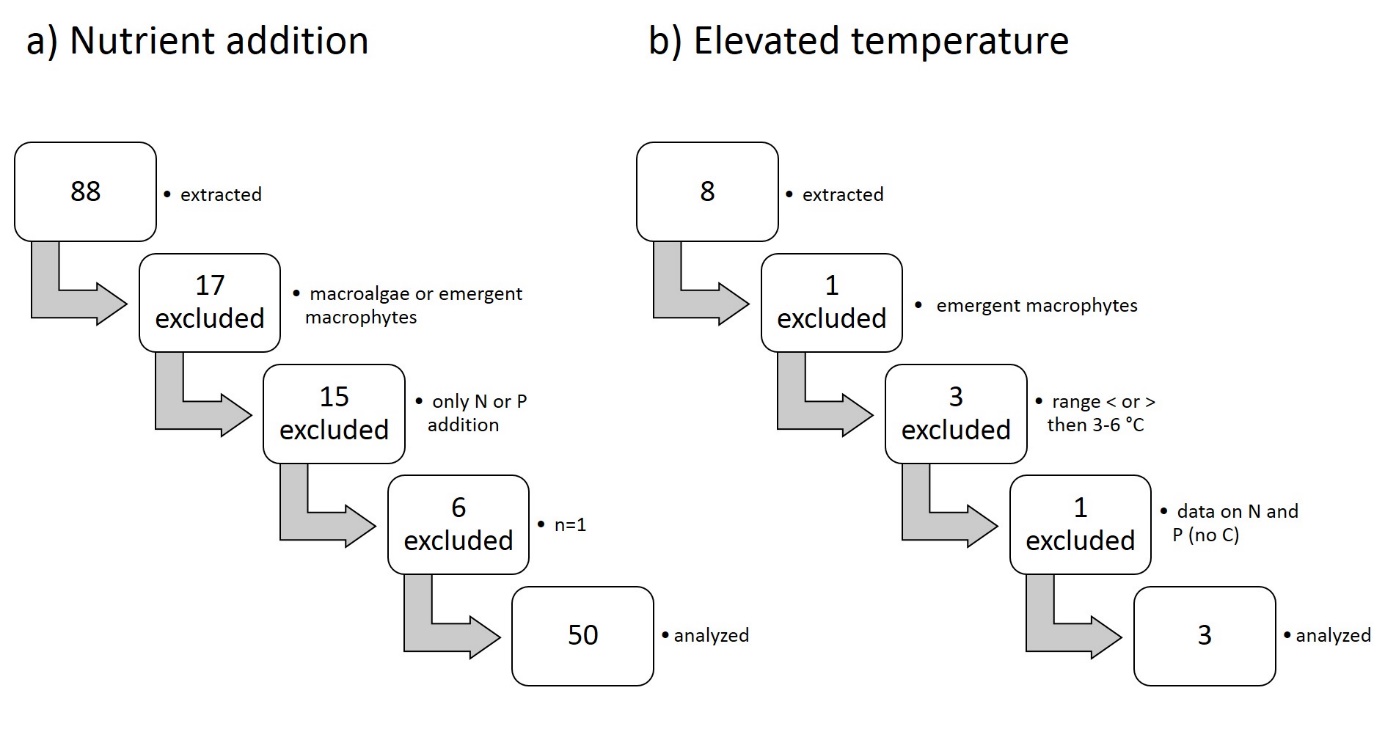


Fig. S2. Stepwise selection process of the studies used in the meta-analysis of nutrient addition, starting with 88 studies (A) and warming, starting with 8 studies (B).
