# Supplementary material for: Impact of Temperature and Nutrients on Carbon: Nutrient Tissue Stoichiometry of Submerged Aquatic Plants: An Experiment and Meta-Analysis
Source: Front Plant Sci. 2017 May 4;8:655. doi: 10.3389/fpls.2017.00655 (PMC5416745; doi:10.3389/fpls.2017.00655)
Supplement: Supplementary file 3 [file DataSheet3.docx]

Supplementary material S3. Overview of papers selected for the meta-analysis (database can be found in Dryad repository)

Anton, A., Cebrian, J., Heck, K. L., Duarte, C. M., Sheehan, K. L., Miller, M. E. C., & Foster, D. (2011). Decoupled effects (positive to negative) of nutrient enrichment on ecosystem services. Ecological Applications, 21(3), 991-1009.

Armitage, A. R., & Fourqurean, J. W. (2006). The short-term influence of herbivory near patch reefs varies between seagrass species. Journal of Experimental Marine Biology and Ecology, 339(1), 65-74.

Armitage, A. R., Frankovich, T. A., Heck, K. L., & Fourqurean, J. W. (2005). Experimental nutrient enrichment causes complex changes in seagrass, microalgae, and macroalgae community structure in Florida Bay. Estuaries, 28(3), 422-434.

Baggett, L. P., Heck, K. L., Frankovich, T. A., Armitage, A. R., & Fourqurean, J. W. (2013). Stoichiometry, growth, and fecundity responses to nutrient enrichment by invertebrate grazers in sub-tropical turtle grass (Thalassia testudinum) meadows. Marine Biology, 160(1), 169-180.

Bakker, E. S., Dobrescu, I., Straile, D., & Holmgren, M. (2013). Testing the stress gradient hypothesis in herbivore communities: facilitation peaks at intermediate nutrient levels. Ecology, 94(8), 1776-1784.

Bakker, E. S., & Nolet, B. A. (2014). Experimental evidence for enhanced top-down control of freshwater macrophytes with nutrient enrichment. Oecologia, 176(3), 825-836.

Cambridge, M. L., & Kendrick, G. A. (2009). Contrasting responses of seagrass transplants (Posidonia australis) to nitrogen, phosphorus and iron addition in an estuary and a coastal embayment. Journal of Experimental Marine Biology and Ecology, 371(1), 34-41.

Castejon-Silvo, I., Terrados, J., Dominguez, M., & Morales-Nin, B. (2012). Epiphyte response to in situ manipulation of nutrient availability and fish presence in a Posidonia oceanica (L.) Delile meadow. Hydrobiologia, 696(1), 159-170.

Ceccherelli, G., & Cinelli, F. (1999). A pilot study of nutrient enriched sediments in a Cymodocea nodosa bed invaded by the introduced alga Caulerpa taxifolia. Botanica Marina, 42(4), 409-417.

Christianen, M. J. A., Govers, L. L., Bouma, T. J., Kiswara, W., Roelofs, J. G. M., Lamers, L. P. M., & van Katwijk, M. M. (2012). Marine megaherbivore grazing may increase seagrass tolerance to high nutrient loads. Journal of Ecology, 100(2), 546-560.

Cronin, G., & Lodge, D. M. (2003). Effects of light and nutrient availability on the growth, allocation, carbon/nitrogen balance, phenolic chemistry, and resistance to herbivory of two freshwater macrophytes. Oecologia, 137(1), 32-41.

Erftemeijer, P. L. A., Stapel, J., Smekens, M. J. E., & Drossaert, W. M. E. (1994). The limited effect of in-situ phosphorus and nitrogen additions to seagrass beds on carbonate and terrigenous sediments in South Sulawesi, Indonesia. Journal of Experimental Marine Biology and Ecology, 182(1), 123-140.

Ferdie, M., & Fourqurean, J. W. (2004). Responses of seagrass communities to fertilization along a gradient of relative availability of nitrogen and phosphorus in a carbonate environment. Limnology and Oceanography, 49(6), 2082-2094.

Han, Q. Y., Soissons, L. M., Bouma, T. J., van Katwijk, M. M., & Liu, D. Y. (2016). Combined nutrient and macroalgae loads lead to response in seagrass indicator properties. Marine Pollution Bulletin, 106(1-2), 174-182.

Hao, B. B., Wu, H. P., Shi, Q., Liu, G. H., & Xing, W. (2013). Facilitation and competition among foundation species of submerged macrophytes threatened by severe eutrophication and implications for restoration. Ecological Engineering, 60, 76-80.

Herbert, D. A., & Fourqurean, J. W. (2008). Ecosystem structure and function still altered two decades after short-term fertilization of a seagrass meadow. Ecosystems, 11(5), 688-700.

Holzer, K. K., & McGlathery, K. J. (2016). Cultivation grazing response in seagrass may depend on phosphorus availability. Marine Biology, 163(4).

Martinez-Crego, B., Arteaga, P., Tomas, F., & Santos, R. (2016). The role of seagrass traits in mediating Zostera noltei vulnerability to mesograzers. Plos One, 11(6).

Martinez-Crego, B., Olive, I., & Santos, R. (2014). CO2 and nutrient-driven changes across multiple levels of organization in Zostera noltii ecosystems. Biogeosciences, 11(24), 7237-7249.

Murray, L., Dennison, W. C., & Kemp, W. M. (1992). Nitrogen versus phosphorus limitation for growth of an estuarine population of eelgrass (Zostera marina L.). Aquatic Botany, 44(1), 83-100.

Olsen, Y. S., & Valiela, I. (2010). Effect of sediment nutrient enrichment and grazing on turtle grass Thalassia testudinum in Jobos Bay, Puerto Rico. Estuaries and Coasts, 33(3), 769-783.

Peralta, G., Bouma, T. J., van Soelen, J., Perez-Llorens, J. L., & Hernandez, I. (2003). On the use of sediment fertilization for seagrass restoration: a mesocosm study on Zostera marina L. Aquatic Botany, 75(2), 95-110.

Perez, M., Romero, J., Duarte, C. M., & Sandjensen, K. (1991). Phosphorus limitation of Cymodecea nodosa growth. Marine Biology, 109(1), 129-133.

Prado, P., Romero, J., & Alcoverro, T. (2010). Nutrient status, plant availability and seasonal forcing mediate fish herbivory in temperate seagrass beds. Marine Ecology Progress Series, 409, 229-239.

Spivak, A. C., Canuel, E. A., Duffy, J. E., & Richardson, J. P. (2009). Nutrient enrichment and food web composition affect ecosystem metabolism in an experimental seagrass habitat. Plos One, 4(10).

Touchette, B. W., Burkholder, J. M., & Glasgow, H. B. (2003). Variations in eelgrass (Zostera marina L.) morphology and internal nutrient composition as influenced by increased temperature and water column nitrate. Estuaries, 26(1), 142-155.

Ventura, M., Liboriussen, L., Lauridsen, T., SØNdergaard, M., SØNdergaard, M., & Jeppesen, E. (2008). Effects of increased temperature and nutrient enrichment on the stoichiometry of primary producers and consumers in temperate shallow lakes. Freshwater Biology, 53(7), 1434-1452.

Xie, Y. H., An, S. Q., & Wu, B. F. (2005). Resource allocation in the submerged plant Vallisneria natans related to sediment type, rather than water-column nutrients. Freshwater Biology, 50(3), 391-402.

Zhang, P. Y., Bakker, E. S., Zhang, M., & Xu, J. (2016). Effects of warming on Potamogeton crispus growth and tissue stoichiometry in the growing season. Aquatic Botany, 128, 13-17.
