# Supplementary material for: Impact of Temperature and Nutrients on Carbon: Nutrient Tissue Stoichiometry of Submerged Aquatic Plants: An Experiment and Meta-Analysis
Source: Front Plant Sci. 2017 May 4;8:655. doi: 10.3389/fpls.2017.00655 (PMC5416745; doi:10.3389/fpls.2017.00655)
Supplement: Supplementary file 5 [file DataSheet5.DOCX]

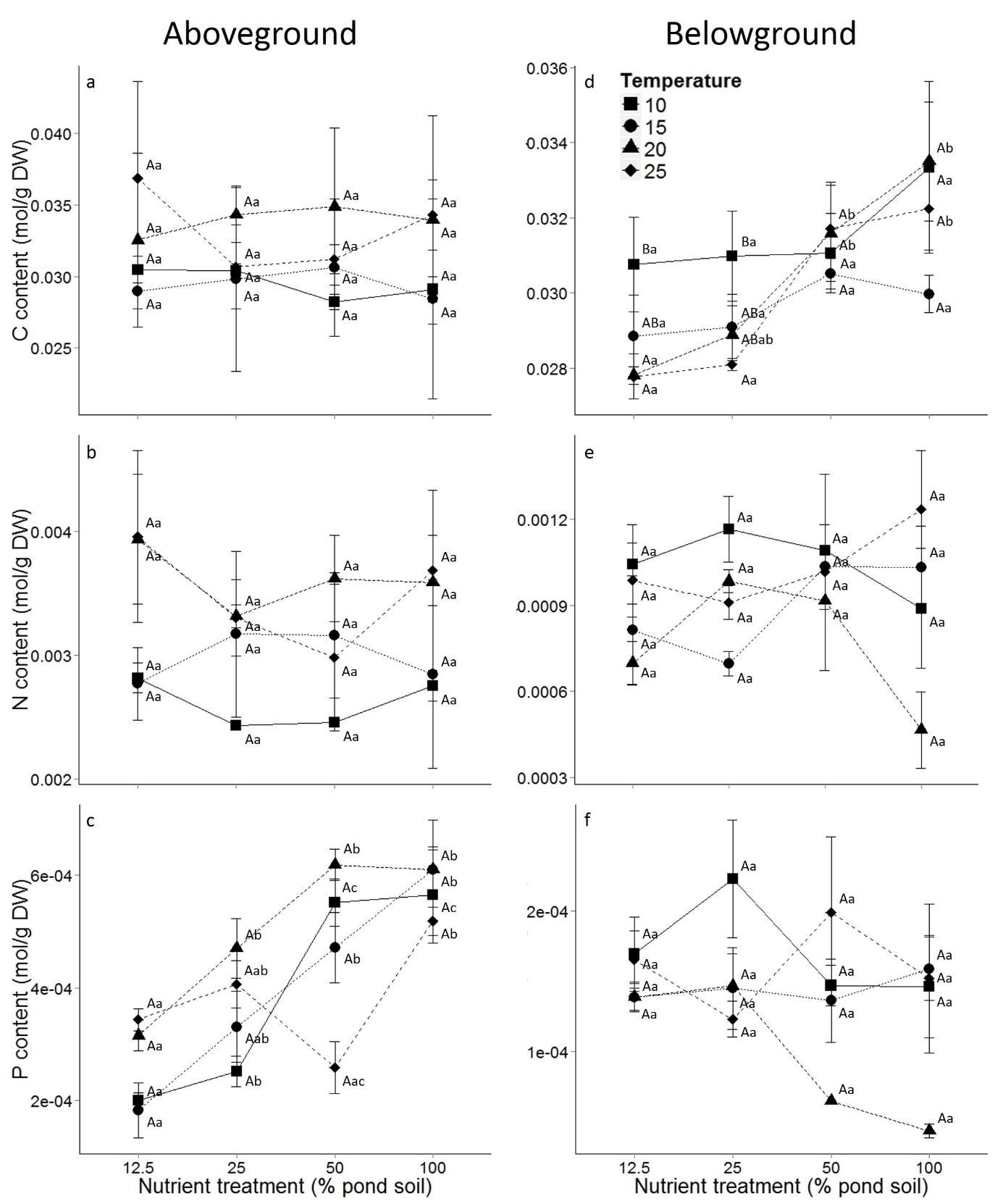


Fig. S5. Above- (A-C) and belowground (D-F) elemental contents of *Elodea nuttallii* in response to sediment nutrient content. Temperature treatments include 10 (■), 15 (●), 20 (▲) and 25 (♦) °C. Dots represent means and error bars standard error of the mean. Capital and lower case letters indicate post-hoc differences between temperature and nutrient treatments, respectively.
