# Supplementary material for: Impact of Temperature and Nutrients on Carbon: Nutrient Tissue Stoichiometry of Submerged Aquatic Plants: An Experiment and Meta-Analysis
Source: Front Plant Sci. 2017 May 4;8:655. doi: 10.3389/fpls.2017.00655 (PMC5416745; doi:10.3389/fpls.2017.00655)
Supplement: Supplementary file 6 [file DataSheet6.DOCX]

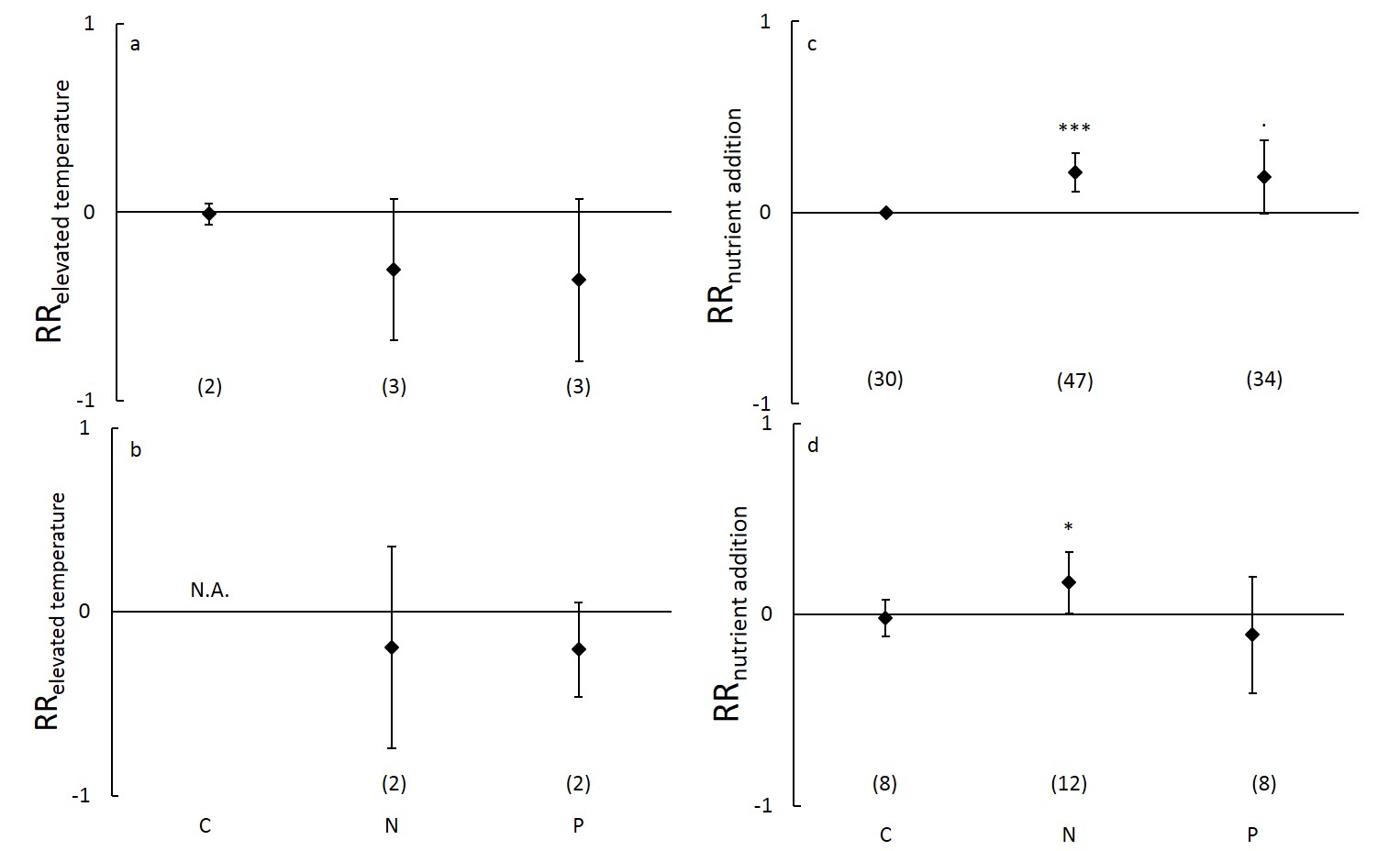


Fig. S6. Natural-log response ratios of C, N and P contents to (A-B) temperature and (C-D) nutrient addition in (A,C) above- and (B,D) belowground biomass of submerged aquatic plants. Bars represent means, error bars 95% confidence intervals, sample size is indicated between brackets and significance levels are indicated as follows: ***:P<0.001, **:P<0.01, *:P<0.05, ^˙^:P<0.10.
