# Supplementary material for: Impact of Temperature and Nutrients on Carbon: Nutrient Tissue Stoichiometry of Submerged Aquatic Plants: An Experiment and Meta-Analysis
Source: Front Plant Sci. 2017 May 4;8:655. doi: 10.3389/fpls.2017.00655 (PMC5416745; doi:10.3389/fpls.2017.00655)
Supplement: Supplementary file 7 [file DataSheet7.DOCX]

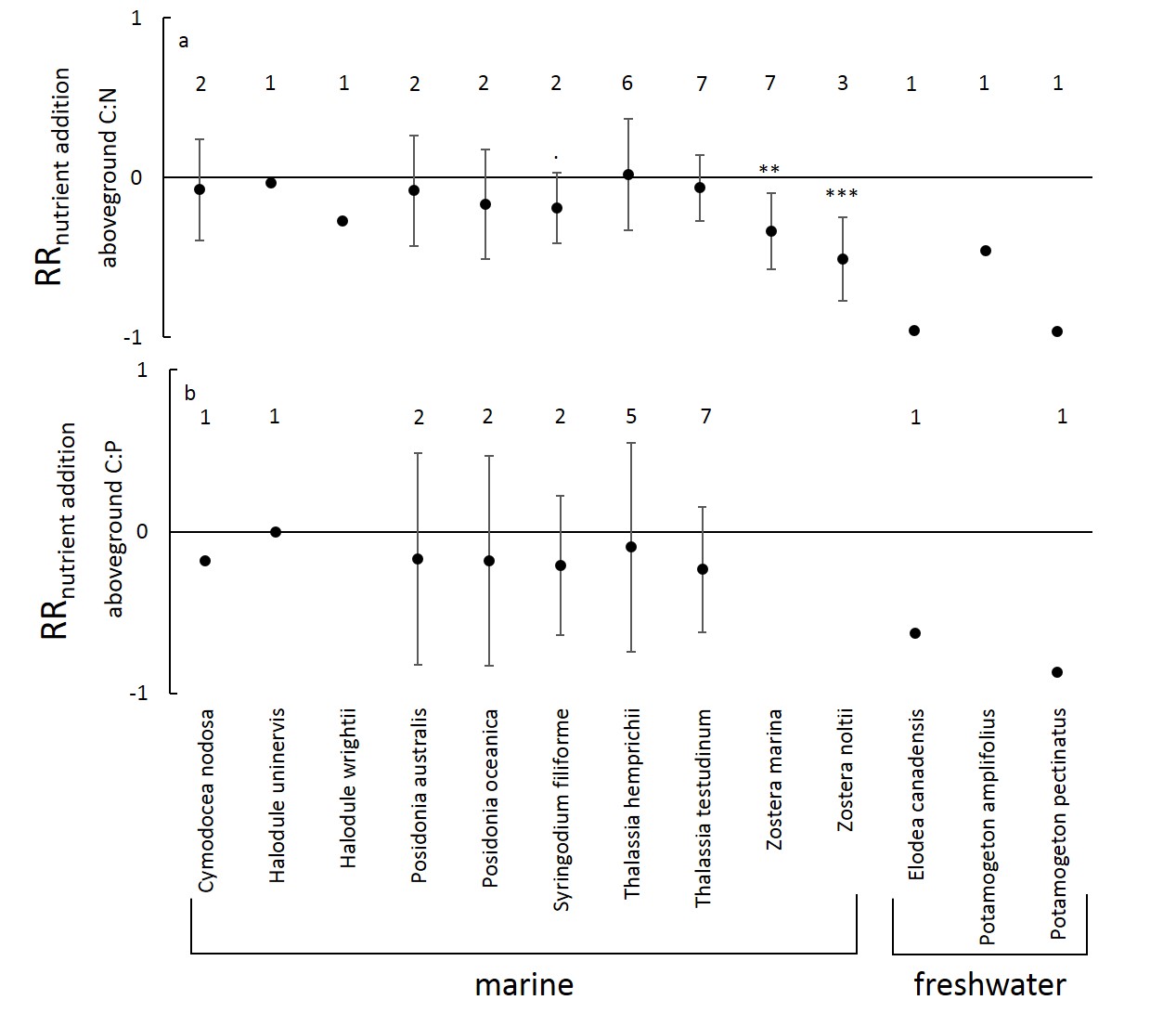


Fig. S7. Species specific natural-log response ratios to nutrient addition in (A) aboveground C:N and (B) C:P ratios. Values represent means, error bars 95% confidence intervals, datalabels sample size, and significance levels are indicated as follows: ***:P<0.001, **:P<0.01, *:P<0.05, ^˙^:P<0.10.
